# A Case Report on Gastric Remnant Intussusception

**DOI:** 10.7759/cureus.21320

**Published:** 2022-01-17

**Authors:** Chase Lazenby, Jeffrey A Nielson, Rebecca S Perry

**Affiliations:** 1 Emergency Medicine, Kettering Health Network, Dayton, USA

**Keywords:** esophagogastroduodenoscopy (egd), emergency medicine, billroth, gastric bypass surgery, intussusception

## Abstract

We present a case of intussusception of a gastric remnant in a patient years after undergoing a Billroth II procedure that was treated with esophagogastroduodenoscopy. Although rare in adults, intussusception has been documented with increasing frequency in adult patients who have undergone Billroth II, mini-gastric bypass, and Roux-en-Y gastric bypass surgery. Timely management can decrease damage due to ischemia.

## Introduction

Intussusception is a presentation of intestinal obstruction observed most often in pediatrics and rarely in adults. Of reported cases of intussusception, adults make up roughly 5%, and of all adult obstructions, 1% is attributed to intussusception [1]. Likewise, not only are there differences in the frequency of this presentation, but treatment modalities and causes differ between adults and pediatrics as well. In pediatrics, the causes are typically benign and treated with air contrast enema [1]. However, most cases of adult intussusception are the result of a secondary cause, typically related to a pathologic condition that creates a leading point like polyps, colonic diverticulum, Meckel’s diverticulum, carcinomas, and strictures, most of which are identified intraoperatively [2]. It is estimated that 70% to 90% of adult cases require definitive treatment, with resection being a common choice [2].

Intussusception has also been documented with increasing frequency in adults who have undergone gastric bypass surgery [3]. This has generally included Billroth II, mini-gastric bypass, and Roux-en-Y gastric bypass. The underlying cause of intussusception after Billroth or gastric bypass is somewhat different from regular causes of intussusception and is speculated to be related to abnormal motility of the divided small bowel [4]. Currently, the preferred imaging modality is computed tomography due to its highest diagnostic accuracy [2].

## Case presentation

**Figure 1:**
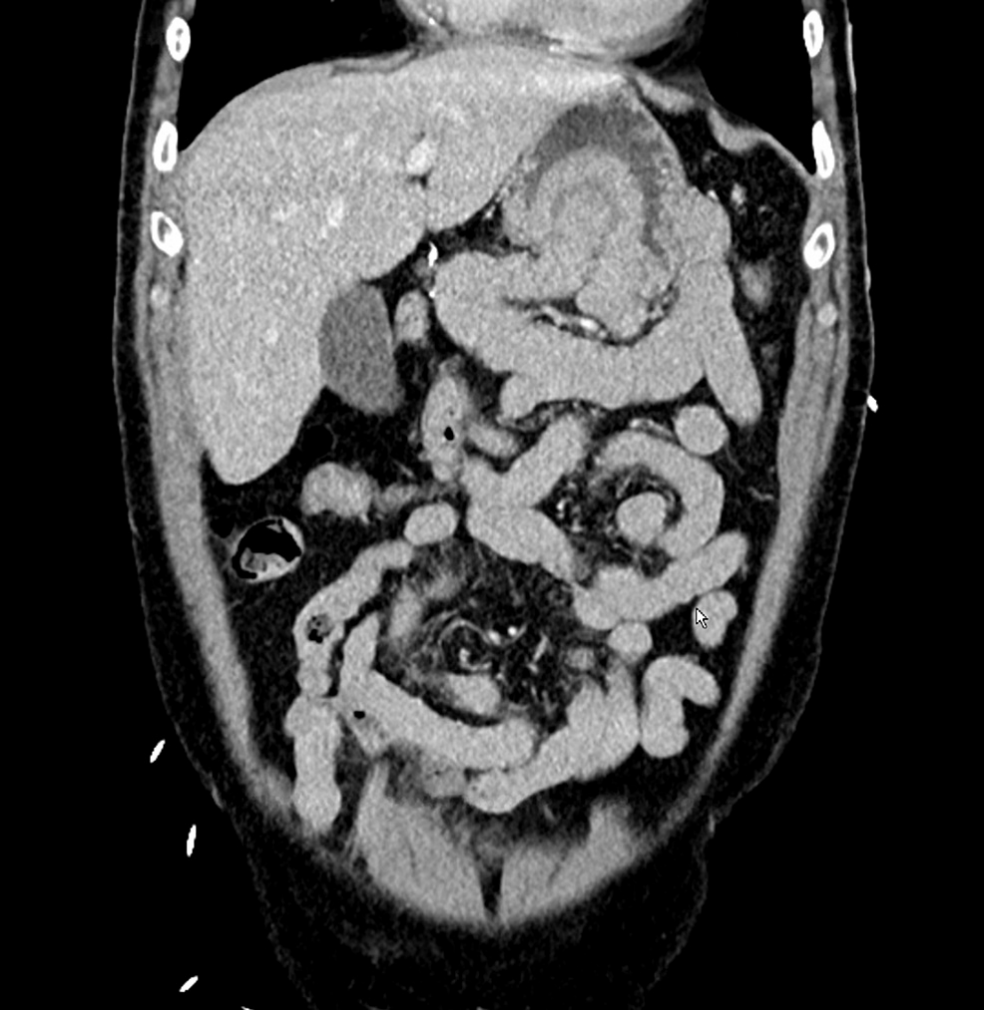
Frontal view of gastric intussusception

**Figure 2:**
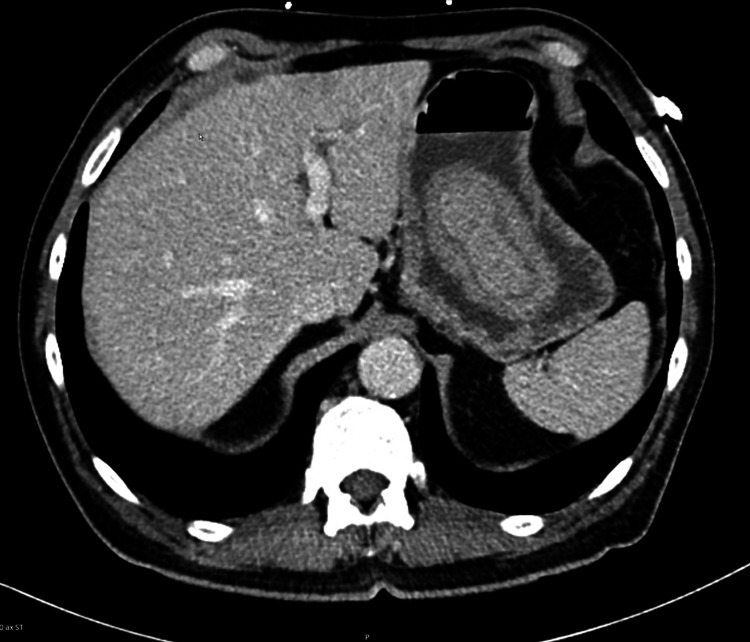
Transverse view of gastric intussusception

A 70-year-old male presented to the emergency department complaining of abdominal pain, diarrhea, nausea, vomiting, and mild hematemesis for about eight hours. The patient’s abdominal pain was mainly located in the epigastrium and left upper quadrant of the abdomen. The patients' past surgical history was positive for a Billroth II partial gastrectomy many years prior after a perforated gastric ulcer. Vital signs in the emergency department included a blood pressure of 153/81 to 159/101, a heart rate peak of 120, a respiratory rate of 11 to 24, a temperature of 96.3, and a SpO_^2^_ of 100%. A physical exam showed a soft but diffusely tender abdomen with localized pain in the epigastrium and left upper quadrant. The patient did demonstrate guarding, and there was a well-healed surgical scar in the left upper quadrant. Abnormal labs included lactic acid of 3 mmoL/L. Normal results were obtained on a complete blood count, complete metabolic count, blood cultures, urinalysis, and lipase, which were within normal limits. A CT abdomen and pelvis were ordered, which demonstrated an intussusception of the proximal small bowel into the gastric bypass remnant without obvious obstruction (Figures 1-2).

General surgery and gastroenterology were consulted. Gastroenterology performed an esophagogastroduodenoscopy and manual reduction was performed with the endoscope in the operating room. The patient made a full recovery and was discharged to go home.

## Discussion

The diagnosis of acute medical and surgical emergencies are some of the hallmarks of emergency medicine. Emergency medicine practitioners consider past medical and surgical history when considering differential diagnoses. This case is noteworthy because it clearly demonstrates the importance of recognizing past surgical history relating to a patient’s current surgical emergency. Gastric bypass surgery is becoming increasingly common, and although early post-operative issues are common, late complications should also be considered, including intussusception, which carries high morbidity.

This case is unique because the patient presented with a soft but diffusely tender abdomen with localized pain in the epigastrium and left upper quadrant. Although there was diffuse tenderness with localized pain, this patient did not have abdominal rigidity or distention. In light of his nausea, vomiting, and diarrhea, it is not impossible to imagine a provider forgoing CT imaging for the possibility of benign gastroenteritis. However, after consideration of the surgical history, presentation, and physical exam, CT imaging should be considered. Delayed diagnosis of intussusception often results in ischemic bowel and perforation. As stated previously, this patient had undergone a Billroth II procedure which resulted in anatomy similar to that of a Roux-en-Y bariatric surgery. This case demonstrates that in the setting of past reconstructive gastric surgery and abdominal pain, the provider should have a lower threshold for imaging. As described above, the preferred imaging modality is computed tomography for the highest diagnostic accuracy [2].

## Conclusions

Intussusception is rare in the adult population but has been documented with increasing frequency in adult patients who have undergone gastric bypass. With the worldwide increase in bariatric surgery, the diagnosis of intussusception has become increasingly more common, and timely management is imperative to avoid any ischemic damage. We report a case of intussusception of a gastric remnant that a patient sustained years after undergoing a Billroth II procedure for a perforated gastric ulcer. Most adults with intussusception require surgical intervention. However, this patient had a successful manual endoscopic reduction.
